# Ethnic and racial characteristics of hemifacial spasm in Hawaii: a clinical cohort analysis

**DOI:** 10.1186/s12883-026-05174-z

**Published:** 2026-07-17

**Authors:** Joo Won Choi, Enrique Carrazana, Richard Ho, Kyle Ishikawa, Hyeong Jun Ahn, Kyung Moo Kim, Eonjung Angeline Kim, Barbara L. Pitts, Kore Kai Liow

**Affiliations:** 1 Department of Neurosciences, UC San Diego School of Medicine, La Jolla, San Diego, CA USA; 2 University of Hawaii at Mānoa, John A. Burns School of Medicine, Honolulu, HI USA; 3 Department of Diagnostic, Molecular, and Interventional Radiology, Icahn School of Medicine at Mount Sinai West, New York, NY USA; 4 Biostatistics Core, John A. Burns School of Medicine, University of Hawaii at Mānoa, Honolulu, HI USA; 5 Department of Psychiatry, John A. Burns School of Medicine, Honolulu, HI USA; 6 Hawaii Pacific Neuroscience, Headache and Facial Pain Center, Honolulu, HI USA

**Keywords:** Hemifacial spasm, Ethnicity, Asian, Native Hawaiian, Pacific islander

## Abstract

**Background:**

Hemifacial spasm (HFS) is frequently reported in Asian populations, yet most prior studies arise from homogeneous ethnic cohorts, limiting our understanding of racial and ethnic disparities as well as potential diagnostic biases. Hawaii’s multiethnic community—which includes large Asian and Native Hawaiian / Pacific Islander (NHPI) populations with regionally high rates of hypertension and other cardiovascular risk factors—offers an opportunity to explore disparities in patients with HFS in a unique epidemiological context.

**Methods:**

We retrospectively analyzed data from 60 HFS patients at a single center and compared racial/ethnic proportions to a clinic-wide cohort of 1,019 neurological patients. Expected counts for each racial group in the HFS cohort were computed from the clinic data, and a chi-square goodness-of-fit test assessed differences between observed and expected counts. Additionally, cardiovascular risk factors in our HFS cohort were evaluated against the statewide population of Hawaii to determine whether these comorbidities were disproportionately represented in the HFS cohort.

**Results:**

Asians comprised 42% of HFS cases but only 23% of the clinic population, while NHPIs represented 30% of cases and 29% of the clinic population. Whites were underrepresented in HFS cases (22%) compared to the clinic population (32%). The chi-square test showed significant deviation from expected clinic proportions (χ² =13.32, df = 3, *p* < 0.01), driven primarily by Asian overrepresentation. In the multivariate analysis adjusting for age and sex, Asian patients had 3.17-fold (*p* = 0.003) higher odds of an HFS diagnosis compared to Whites.

**Conclusion:**

Within a multiethnic neurologic clinic, Asian patients, particularly Asian women, are significantly overrepresented among those affected by HFS, consistent with prior work. These findings underscore the need for further research into genetic, anatomic, hormonal, or healthcare-related contributors to these disparities, especially in ethnically diverse regions like Hawaii.

## Introduction

Hemifacial spasm (HFS) is an uncommon movement disorder characterized by involuntary facial muscle contractions [1]. A higher Asian prevalence has been suggested; however, these observations arise largely from populations and clinical practices that may not mirror the community diversity found in Hawaii [2, 3]. Indeed, both Asian ancestry and hypertension, an established risk factor for neurovascular disorders, are common in Hawaii’s population, creating an opportunity to examine patterns of HFS presentation in a diverse clinical setting [4]. While data on other groups are limited, most large clinic-based and population studies from Europe and North America describe predominantly White cohorts, with relatively few cases reported among Black, Hispanic, or Latino patients, suggesting possible ethnic variation in risk or ascertainment [5, 6]. Our clinic cohort study leverages this setting, contrasting the observed distribution of HFS cases among different ethnic groups relative to the demographic composition of patients seen in our general neurology clinic.

## Methods

We conducted a retrospective chart review of all adult patients aged ≥ 18 who had an active diagnosis of HFS at a large neuroscience institute in Hawaii between January 1, 2010, and January 1, 2021. Patients with HFS were identified using the International Classification of Diseases Ninth or Tenth Revisions, Clinical Modification (ICD-9-CM or ICD-10-CM) codes 351.8 and G51.3x. All HFS diagnoses were confirmed by in-house neurologists. A clinic-wide control cohort was chosen from our institution’s patient pool consisting of 1,019 randomly selected patients spanning the same time range during which our HFS patients were selected. These records were filtered to ensure they represented unique individuals, excluding any repeat longitudinal visits or duplicate entries. Patient demographic data, including self-identified race/ethnicity, were collected from medical records. The study was approved by the University of Hawaii John A. Burns School of Medicine Institutional Review Board (IRB #2020 − 01010), with waiver of informed consent for retrospective de-identified data analysis.

Expected counts of HFS cases in each racial category were calculated by multiplying the proportion of each race in the clinic population by the total number of HFS cases (60). A chi-square goodness-of-fit test was performed to assess whether the observed racial distribution of HFS cases significantly deviated from expected counts based on the clinic population distribution. The test statistics were calculated as

$$\:X^2\:=\:{\sum\:({\mathrm{O}}_{\mathrm{i}\:}-\:{\mathrm{E}}_{\mathrm{i}})}^{2}\:/\:{\mathrm{E}}_{\mathrm{i}}$$

where Oi and Ei are observed and expected counts, respectively, for each racial group. The degrees of freedom were set to the number of categories minus one (df = 3). A p-value < 0.05 was considered statistically significant. We also conducted univariate and multivariable logistic regression analyses, incorporating Firth’s correction, to identify independent variables associated with HFS [7]. All tests employed a two-tailed approach with a significance level set at *p* < 0.05. Statistical analyses were conducted using SPSS version 29.0 (IBM Corp., Armonk, NY, USA). For analyses of race and ethnicity, the neurological clinic population served as the reference group, as it reflects the underlying demographic composition of patients from which HFS cases were drawn.

To place cardiovascular comorbidity patterns in context, we compared the prevalence of hypertension and obesity in the HFS cohort with published population-level estimates from Hawaii. This approach was chosen to mitigate potential bias since many neurological conditions seen in the clinic are strongly associated with cardiovascular risk factors, which could inflate baseline rates of hypertension and obesity in the clinical population. We used statewide benchmarks from reputable sources such as the Behavioral Risk Factor Surveillance System (BRFSS) and Hawaii Department of Health reports, which provide race-specific prevalence data for hypertension, obesity, and mean BMI among Asian, White, and Native Hawaiian / Pacific Islander (NHPI) adults. Using Hawaii’s 2025 population estimates and these prevalence ranges, we calculated expected case counts for each racial group scaled to match our study sample size. We then applied chi-square goodness-of-fit tests to compare observed versus expected counts within each racial category. This allowed us to assess whether cardiovascular risk factors were disproportionately represented in the HFS cohort compared to population-based expectations. Statistical significance was set at *p* < 0.05.

In addition, hypertension and obesity prevalences among HFS patients by race were compared to expected prevalence derived from the broader neurology clinic cohort, to ascertain whether differences between HFS and other neurological diagnoses would emerge. Observed and expected counts were analyzed using chi-square goodness-of-fit tests to assess whether cardiovascular comorbidities differed significantly by race. Statistical significance was set at *p* < 0.05.

## Results

The HFS cohort comprised 60 patients with the following racial composition: 42% Asian (*n* = 25), 30% NHPI (*n* = 18), 22% White (*n* = 13), and 7% Other (*n* = 4). Female predominance was observed overall, with 72% (*n* = 43) of the cohort being female. Gender distribution varied by race, with 96% (*n* = 24) of Asian patients being female, 56% (*n* = 10) of NHPI patients being female, and 69% (*n* = 9) of White patients being female. Median age of HFS diagnosis across the entire cohort was 53 years; Asians were diagnosed at a median age of 67 years, NHPIs at 54 years, and Whites at 41 years. Median time to diagnosis from symptom onset was longest in Asians (36 months), compared to 12 months for Whites and NHPIs. These results are summarized in Table 1. Comparatively the general neurological clinic population (*n* = 1,019) was 23% (*n* = 232) Asian, 29% (*n* = 296) NHPI, 32% (*n* = 322) White, and 17% (*n* = 169) Other.

**Table 1:** Descriptive characteristics of hemifacial spasm patients

Characteristic	Race / Ethnicity		
	White (22%), *N* = 13	Asian (42%), *N* = 25	NHPI (30%), *N* = 18
Sex			
Female	9 (69%)	24 (96%)	10 (56%)
Male	4 (31%)	1 (4.0%)	8 (44%)
Age^1^	51 (44, 56)	67 (60, 78)	54 (45, 64)
Obesity	3 (23%)	4 (16%)	7 (39%)
Hypertension	1 (7.7%)	11 (44%)	9 (50%)
Time to HFS Diagnosis^1^ (Months)	12 (5, 24)	36 (20, 48)	12 (3, 24)
Age at HFS Diagnosis^1^	41 (39, 49)	63 (55, 70)	50 (37, 59)
Time to HFS Treatment^1^ (Months)	4 (1, 10)	36 (3, 48)	3 (1, 15)

^1^Median (25% Quartile, 75% Quartile)

The chi-square goodness-of-fit test revealed that the observed racial distribution of HFS patients significantly differed from the expected distribution based on the clinic population proportions (χ² = 13.32, degrees of freedom = 3, *p* < 0.01). This significant deviation was primarily driven by the overrepresentation of Asians and the underrepresentation of Whites and Other racial groups among HFS cases. These statistical findings are summarized in Table 2.

**Table 2:** Comparison of observed hemifacial spasm cases to expected counts based on clinic population racial distribution. The chi-square test statistic is 13.32 with 3 degrees of freedom, which corresponds to a p-value less than 0.01, indicating a statistically significant difference between observed and expected racial distribution of HFS cases within the clinic population

Race / Ethnicity	Observed (HFS Cases, O_i_)	Expected (Clinic Population, E_i_)	(O_i_ − E_i_)^2^ / E_i_
White	13	19	1.8947
Asian	25	14	8.6429
NHPI	18	18	0.0000
Other	4	9	2.7778
Total	60	60	13.3154

In our multivariate analyses, when controlling for age and race, HFS patients were significantly less likely to be male (OR: 0.37, 95% CI: 0.18, 0.72; *p* = 0.005). After adjusting for sex and age, Asian and NHPI patients had a 3.17 (95% CI: 1.51, 6.95; *p* = 0.003) and 2.73 (95% CI: 1.23, 6.22; *p* = 0.015) fold higher odds of having an HFS diagnosis compared to Whites. There was no significant difference in age between the HFS and the clinic cohorts in both univariate and multivariate analyses. These findings are outlined in Table 3.

**Table 3:** Univariate and multivariate logistic regression of hemifacial spasm patients compared to controls

Characteristic	Unadjusted Odds Ratios			Adjusted Odds Ratios		
	OR^1^	95% CI^1^	*p*-value	OR^1^	95% CI^1^	*p*-value
Sex						
Female	—	—		—	—	
Male	0.39	0.20, 0.75	**0.006**	0.37	0.18, 0.72	**0.005**
Age	1	0.98, 1.01	0.6	0.99	0.98, 1.01	0.4
Race						
White	—	—		—	—	
Asian	2.91	1.42, 6.24	**0.004**	3.17	1.51, 6.95	**0.003**
NHPI	2.74	1.26, 6.14	**0.012**	2.73	1.23, 6.22	**0.015**
Other	1.86	0.48, 6.02	0.3	1.76	0.45, 5.81	0.4

To contextualize cardiovascular risk factors in our cohort, we compared the prevalence of hypertension and obesity in HFS patients with published population-level estimates from Hawaii. Specifically, statewide survey data (BRFSS and Department of Health reports) indicate that hypertension affects ~ 20–25% of Asian adults, ~ 25–30% of White adults, and ~ 25–40% of NHPI adults [8]. In contrast, 44% (*n* = 11) of Asian HFS patients and 50% (*n* = 9) of NHPI HFS patients in our cohort reported hypertension, while only 7.7% (*n* = 1) of White HFS patients had hypertension. With respect to obesity, general population prevalence is ~ 13–19% in Asians, ~ 19–20% in Whites, and > 40% in NHPIs. By comparison, obesity was noted in 16% (*n* = 4) of Asians, 23% (*n* = 3) of Whites, and 39% (*n* = 7) of NHPI HFS patients.

Asians with HFS demonstrated significant disparities in hypertension prevalence (χ² range 4.81- 9.00, *p* < 0.05). NHPI with HFS had comparable rates of hypertension and obesity compared to expected values based on statewide prevalence. Whites showed lower hypertension but higher obesity representation, though not statistically significant. These findings are listed in Table 4.

**Table 4:** Comparison of observed versus expected cases of hypertension and obesity by race/ethnicity in the hemifacial spasm cohort relative to Hawaii population-based prevalence estimates (sample-scaled chi-square analysis, n varies by group)

Characteristic	Race / Ethnicity	HFS cases with comorbid hypertension or obesity	Expected *n* (range)	χ² Statistic (range)	*p*-value
Hypertension	Asian	11 / 25 (44.0%)	5.00–6.25	4.81–9.00	0.0027–0.028
	NHPI	9 / 18 (50.0%)	4.50–7.20	0.75–6.00	0.014–0.386
	White	1 / 13 (7.7%)	3.25–3.90	2.08–3.08	0.079–0.149
Obesity	Asian	4 / 25 (16.0%)	3.25–4.75	0–0.20	0.655–1.0
	NHPI	7 / 18 (40.0%)	~ 7.2	0.009	~ 0.924
	White	3 / 13 (20.0%)	2.47–2.60	0.077–0.140	0.708–0.781

Significant differences in hypertension and obesity prevalence were observed among racial groups in the HFS cohort compared to expectations based on the neurology clinic population. Asians showed higher hypertension prevalence than expected (χ² = 4.17, *p* = 0.041), while Whites exhibited significantly lower obesity prevalence (χ² = 11.53, *p* = 0.0007). Other comparisons did not reach statistical significance.

## Discussion

Consistent with prior studies, our clinic cohort study showed a higher risk of HFS in patients of Asian descent. Moreover, the broader and more diverse population we examined allowed us to identify significant age- and sex-related distinctions that earlier studies could not capture. Prior reports, such as that by Wu et al., highlighted elevated HFS prevalence among Asians by comparing them to disease-based control groups, but their limited diversity made it difficult to distinguish biological risk from demographic background [2]. Similarly, the study by Au et al. in Singapore detailed the clinical characteristics and psychosocial impact of HFS in a largely Asian cohort, demonstrating common features such as female predominance, mean age of onset around 48 years, initial involvement of the orbicularis oculi muscle, and significant patient-reported stress aggravation [3]. They also highlighted delays in treatment initiation and the effective response to botulinum toxin therapy. However, their scope focused on patient presentation and perceptions within a relatively homogeneous population [3].

By analyzing a neurologic clinic cohort within the context of Hawaii’s unique multiethnic population, including NHPI whose representation aligns with census data, we found that Whites were less frequently represented among HFS cases than expected. Incorporating cardiovascular risk factors and demographic variables, our study provides a more comprehensive view of ethnicity, age, sex, and vascular health influences in HFS, reflecting the complex interplay of these factors in Hawaii’s diverse community.

There are several possible hypotheses for this ethnic predisposition. Prior anatomical differences among Asians have been described, including narrower posterior fossa volumes and altered vertebrobasilar artery dominance and lateralization—both of which increase the risk of neurovascular compression at the facial nerve root exit zone [9, 10]. In addition, the particularly higher rates of hypertension in Asians with HFS than expected based on population prevalence, as demonstrated in our study, likely contribute mechanistically to the pathophysiology of HFS, as chronic hypertension poses increased risk of arterial ectasia and tortuosity which in turn may lead to pulsatile irritation of an adjacent facial nerve [11, 12].

Remarkably, in our sample, nearly all Asian HFS patients (96%) were women, diagnosed at older ages compared to NHPI or White HFS patients. Epidemiological work in Finland and other settings consistently found a higher HFS prevalence in women but these differences have yet to be explored in multiethnic U.S. populations [13, 14]. Such female predominance remains poorly explained, as noted by Nurminen and others, who speculate about social or treatment-seeking differences [7, 13]. It remains plausible that smaller posterior fossa CSF volumes could explain the female preponderance in HFS as well [15].

Our study has several limitations. First, given the single-center, retrospective nature of our study, our relatively small HFS cohort does not encapsulate the true population-level prevalence of HFS diagnosed across Hawaii. In addition, as a tertiary referral center, our HFS cohort is susceptible to certain selection biases, including referral bias, geographical barriers, and disparities in healthcare utilization. While evaluating our cases against an internal control cohort drawn from the same neurology clinic provides a direct comparator for overrepresentation within our institutional pool, it remains an imperfect proxy that cannot resolve external access biases. Second, the way race and ethnicity were recorded relied on patient self-identification or medical records, which may not perfectly reflect genetic ancestry or the complex social factors that also shape health outcomes. Third, to understand cardiovascular risk factors like hypertension and obesity, we compared our patients to statewide data from public health surveys. While this helped provide a fair frame of reference, it is important to note that this external data came from different populations and methods, so caution is needed in comparing them directly. Finally, our study could not explore other important contributors, such as detailed socioeconomic status, comorbid health conditions, or genetic and hormonal influences, which are worthwhile avenues for future prospective research to better explain ethnic differences in HFS.

## Data Availability

The datasets used and/or analyzed during the current study are available from the corresponding author on reasonable request.

## Abbreviations

BMI: body mass index
BRFSS: Behavioral Risk Factor Surveillance System
CSF: cerebrospinal fluid
HFS: hemifacial spasm
ICD-9-CM: International Classification of Diseases, Ninth Revision, Clinical Modification
ICD-10-CM: International Classification of Diseases, Tenth Revision, Clinical Modification
IRB: Institutional Review Board
NHPI: Native Hawaiian / Pacific Islander
